# Synopsis of Discussions at Fourth Annual Meeting of the M. V. A. D. S.

**Published:** 1848-10

**Authors:** 


[Reported for the Dental Register.

### A SYNOPSIS OF DISCUSSIONS,

### At Fourth Annual Meeting of Mississippi Valley Association of Dental Surgeons.

After the reading of Dr. B. B. Brown's paper on Hæmorrhage, Dr. James Taylor remarked, that he had met with several cases of protracted hæmorrhage in the course of his practice. One family in particular, in which he operated, he would notice. In it there had existed for a length of time a hæmorrhagic diathesis. An uncle of the family had died some years ago of hæmorrhage. There are seven sons in this family, but all are not subject to this malady. Four of them are predisposed to hæmorrhage, while the others are not. When any of the former had a tooth extracted, the hæmorrhage would generally cease in the common time, but in two or three days after, it would return. The effects of the various local applications,

(actual cautery, styptic, and pressure,) were tried. The hæmorrhage could thus be controlled for a time, but in ten hours the blood would again flow. This state of things would continue for about ten days, when the hæmorrhage would cease. In the interim the proper agents designed to have a constitutional effect, had been made use of, and these, combined with the diminished force of the blood, by so great a loss, doubtless had been the most efficient means in these instances in arresting the hæmorrhage.

Upon the suggestion of some member, the Doctor said it was his intention, when called upon again to extract a tooth for either of these individuals, to prepare the system by previous constitutional treatment, in order to ascertain if the danger could thus be diminished. In applying pressure in these cases, the Doctor did not confine it to the alveolar cavity, but he made a plate to fit the gums for some distance around it, under which he placed cotton, and this pressure, he thought, had a tendency to draw the edges of the wound together.

Dr. Bonsall cautioned the members in the use of the oil of ergot, (mentioned by Dr. Brown,) in particular stages of female life.

Dr. Berry advised the use of tinct. ferri muriatis, as a styptic, had used it in a recent case of several days standing, with entire success.

During the morning session of the second day, a paper was read by Dr. Bonsall, on Pivot Teeth, which gave rise to a very amicable and interesting discussion.

Dr. Griffith led the way by remarking that in inserting pivot teeth he pursued a course not pointed out in this paper, he had seventeen years ago introduced an improvement in the shape of the pivot, for which he would not say he was entitled to credit, that was for the members of the Society to decide upon. His mode of practice was, (with the exception of persons leaving the city immediately,) always to insert a temporary pivot, fitting easily so as to allow of its ready extraction. In that portion of the pivot which is lodged in the fang, he cut a small groove in one side, so as to form a canal in it to allow the discharge of pus if it formed above the pivot. In from four to fifteen days if no inflammation existed, he inserted the permanent pivot, which he formed of well seasoned hickory. The first individual he informed of his making use of this canal for the egress of pus, was Dr. Stringfellow, who, he understood, varied the practice by filing a groove in the side of the fang, instead of cutting it in the pivot. This mode he could not approve of, as he thought it weakened the root.

Dr. James Taylor remarked that pivoting teeth was undoubtedly contrary to sound physiological principles, as it was a rule in surgery to remove every dead member. But it is a question whether the disadvantages arising from the wearing a pivot tooth, were not less than those growing out of the use of a single plate tooth. He thought that in general they were. The injuries resulting to the teeth bearing the clasps were in many instances quite serious. When but one plate tooth was needed in a mouth he advised that it be dispensed with, unless in the front of the mouth.

Dr. Goddard stated that when applied to for the insertion of a pivot tooth, he always warned the patient that he might expect trouble from the root after the tooth was inserted, as in eight cases in ten an abscess would form. He never used a temporary pivot, but inserted the tooth permanently at once, he never formed a canal alongside his pivot for the egress of pus, and never used a pivot entirely of wood, but always gold inserted in wood, which he condensed by driving the pivot through a hole of the exact size of the drill. He had used gutta percha several times in pivoting teeth, but was not yet prepared to give an opinion on it.

Dr. E. Taylor informed the Society that in this operation he made little use of excising forceps. For the removal of the remaining portion of a crown he prefers a very fine saw, a description of which he had given in the Register. His experience since that period, had confirmed his opinion of its advantages. He had used the wood pivot with and without the canal. When he anticipated trouble, he made use of the canal, has formed this sometimes by cutting off a portion of the pivot, at others has cut a groove or canal in the fang. Has used the gold incased in wood, when the pivot requires an inclination, in other cases commonly uses the hickory pivot. He has also plugged up the cavity above the point of the pivot. He has tried the gutta percha between the crown and the fang, when there has been a saucer shaped cavity surrounding the pivot and thinks it useful in such cases.

Dr. Berry was of opinion that it was best to insert a temporary pivot. The hickory he thought strong enough when the proper kind was selected. He had used some for the last three years which his friend, Dr. James Taylor, had obliged him with, and which is certainly the best he ever used. He thought that which is best suited for pivots, should not be very flexible, but at the same time, should be equally tough with that which was. The pivot should be cut from the growth of one year, so as to avoid the porous fibre between the layers formed in consecutive years.

Dr. Leslie inquired of the older members of the Society, what they had found to be the best means of *allaying* the inflammation which so frequently followed the pivoting method of inserting teeth. In his practice, he always after inserting a pivot tooth, desired the patient to give him immediate notice should the inflammation supervene, in which case he exhibited the saline purgatives, accompanied with a local cooling application, such as—

℞ Sulph. Zinc, ℨss.

Aqua Font, oj.

M.

This to be applied immediately over the root, by means of three or four folds of cotton cloth, which are to be re-wet as the cloth becomes heated. This mode of treatment he had found during his own short experience, entirely successful. In every instance he was enabled to reduce the inflammation by the process of resolution.

Dr. Curtis asked whether a fang which had once suppurated was likely to do so again, or have an abscess formed over it should the crown be re-set. He also wished to know if an abscess ever entirely cicatrized.

Dr. James Taylor remarked, that he had worn pivot teeth for fifteen years, and he found that the gum over the fangs on which they were set, was liable to suppurate once a year, but for several years together he had prevented the formation of an abscess by the timely use of an emetic. He, however, had several times, an abscess form over the same fang, owing to his too long neglecting the usual remedies. He was not prepared to say that an abscess did ever entirely close up, he, however, had one which had not discharged for years. The Doctor stated he had made frequent use of the means spoken of by Dr. L., for allaying inflammation arising from this operation, and he was pleased to say he had done so with success. In answer to an enquiry from the chair, as to what was the proper age a tree should be when cut, and the proper season to cut it in, so as to adapt it best for pivots, the Doctor remarked that he did not consider the age of the tree, or the season it was cut in, of as much importance as the circumstances under which it was grown.—That which his friend Dr. B., had referred to, he was fully satisfied was the best hickory he had ever used. It had been selected by a friend while on a visit to the Allegheny mountains. The directions he gave him was, to go to the south side of a hill, and select a tree which was growing on a barren soil, and which was scrubby and stunted, and from it, cut out a block near the root.

Dr. Taft enquired what was the opinion of the Society regarding the cause of an abscess following the insertion of a pivot tooth. Was it the result of the operation viewed as a whole, or was it the result of a particular portion, or of several parts of the operation? Was it the destruction of the nerve, the excision of the crown, or the drilling of the fang? He thought it was the preparation of the fang.

Dr. Goddard considered the cause of abscess to be a lodgment of foreign matter above the pivot, hence, he was very particular in removing the chips resulting from the formation of the pivot hole. He thought it very important to extract all the nerve. In his own practice he had very rarely found abscess to follow this operation.

Dr. Griffith thought the drill used by him well calculated to keep the cavity free from chips. The head or cutting portion was from an eighth to a quarter of an inch in length, this was formed of four square steel, (the shaft being of a less size,) the flat on each side was cut out with a half round or oval file, so that the instrument when finished had the four angles formed into four cutting surfaces. The chips escaped without difficulty by the groove thus formed on each side, he always used a syringe to clean out the cavity. In view of Dr. C.'s query, he would relate a case in which he had inserted a pivot tooth nine years ago. In this instance he made use of the temporary pivot, and directed the lady to inform him if the inflammation became serious over the fang. This she neglected, and he was not called until after an abscess had formed. After the inflammation had entirely subsided, he inserted the tooth with a permanent pivot, since which she has not suffered from that tooth. He approved of the course pursued by Dr. L., in the early stage of inflammation, but in addition advised the extraction of the pivot, and the perforation, if possible, of the foramen of the fang with a bristle, in order that if pus had formed, it might thus escape.

Dr. McCullum approved of the plugging the fang above the point of the pivot. He had pursued the practice for years, and thought it highly beneficial.

A short and interesting discussion arose upon the question, "Are the teeth of the Negro less liable to decay under the same circumstances, than the teeth of the White."

Dr. Whitney stated that his attention had been early called to the consideration of this question. He had when a student of medicine in Virginia examined it. His attention had also been given it while practising dentistry in northern New York, and lately in Tennessee, and he believed the facts would show that the teeth of the negro were more liable to decay than those of the other class, under similar circumstances. In northern New York, he thought the teeth of the negro were not so good as those he had met with in Tennessee, and certainly not as good as those of the white in New York, living on the same diet. This difference he attributed to the inability of the negro to stand the cold climate of the north.

Dr. E. Taylor considered the difference between the two classes to be governed by the constitution of each. In the north the teeth of the white would be better than those of the black, because the northern climate was not suited to the complete development of the latter. In the south the state of things would be reversed. The impression on the public mind, that the teeth of the blacks were better than those of the whites, had its origin more in the striking contrast between the teeth and the skin, than in a knowledge of the actual state of the teeth of this class.

Dr. Berry differed from his friend, Dr. W., in regard to the liability of the teeth of the negro to decay at the north, he believed it was too cold there six months in the year, for the process of decay to proceed. He thought the teeth of the black at the south just as liable to decay as those of the white. He had examined the mouths of five hundred negroes at the south, and of three hundred of these under the age of thirty years, he had not found more than twenty perfect sets of teeth. The cause of this he believed to be the amount of swine's flesh they eat. The whites although much more careful of and cleanly with their teeth, lost them by the undue use of coffee, pickles, and the like, which were more injurious to the teeth than the swine's flesh, and altogether unfit for the food of man. He said he had never examined the teeth of the mulatto, a race which should never have been allowed to exist on the face of God's earth, but he should infer that they must be worse than either of the other classes.

Dr. Griffith remarked that he had practiced in Virginia, Georgia, and Kentucky, in the whole some nineteen years. His conviction, formed on that experience, is, that the teeth of the blacks were not constitutionally better than those of the whites. The reason their teeth decay as much as they do, with their generally simple diet, must be charged to their careless habits—few of them ever carrying a toothpick.

Dr. Whitney objected to the position of Dr. Berry, regarding the effect of animal diet, and cited the use made of meat by the aborigines of this country, coupled with the perfect state of their teeth, as proof of its incorrectness. A. M. L.

